# Kinetics of Volatile and Nonvolatile Halide Perovskite Devices: The Conductance-Activated Quasi-Linear Memristor (CALM) Model

**DOI:** 10.1021/acs.jpclett.4c03132

**Published:** 2024-12-19

**Authors:** Agustín Bou, Cedric Gonzales, Pablo P. Boix, Yana Vaynzof, Antonio Guerrero, Juan Bisquert

**Affiliations:** † Chair for Emerging Electronic Technologies, Technical University of Dresden, Nöthnitzer Str. 61, 01187 Dresden, Germany; ‡ Leibniz-Institute for Solid State and Materials Research Dresden, Helmholtzstraße 20, 01069 Dresden, Germany; § Institute of Advanced Materials (INAM), Universitat Jaume I, 12006 Castelló, Spain; ∥ Instituto de Tecnología Química (Universitat Politècnica de València-Agencia Estatal Consejo Superior de Investigaciones Científicas), Av. dels Tarongers, 46022, València, Spain

## Abstract

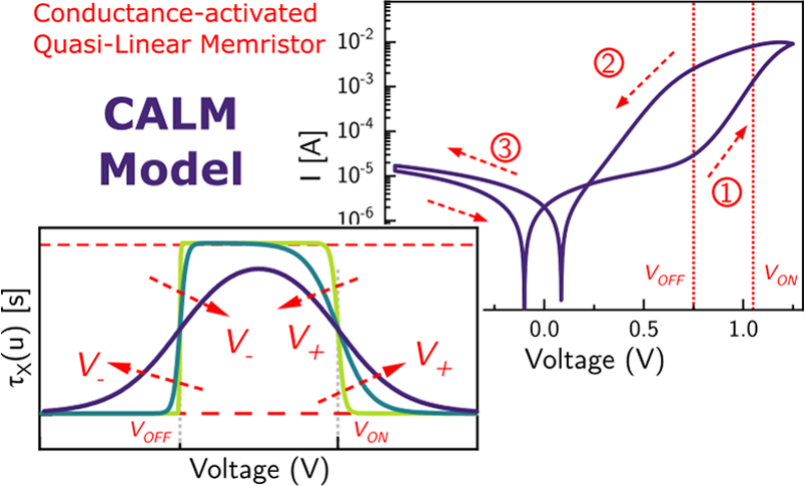

Memristors stand out as promising components in the landscape of memory and computing. Memristors are generally defined by a conductance mechanism containing a state variable that imparts a memory effect. The current–voltage cycling causes transitions of conductance, which are determined by different physical mechanisms, such as the formation of conducting filaments in an insulating surrounding. Here, we provide a unified description of the set and reset processes using a conductance-activated quasi-linear memristor (CALM) model with a unique voltage-dependent relaxation time of the memory variable. We focus on halide perovskite memristors and their intersection with neuroscience-inspired computing. We show that the modeling approach adeptly replicates the experimental traits of both volatile and nonvolatile memristors. Its versatility extends across various device materials and configurations, as W/SiGe/a-Si/Ag, Si/SiO_2_/Ag, and SrRuO_3_/Cr-SrZrO_3_/Au memristors, capturing nuanced behaviors such as scan rate and upper vertex dependence. The model also describes the response to sequences of voltage pulses that cause synaptic potentiation effects. This model is a potent tool for comprehending and probing the dynamical response of memristors by indicating the relaxation properties that control observable responses.

In pursuing the advance of computational paradigms, exploring novel technologies has become a cornerstone in shaping the future of electronic devices.^1^ Among the different computational breakthroughs, resistive random-access memories (ReRAMs), or memristors, have emerged as a promising candidate, presenting an unparalleled blend of memory and neuromorphic computing capabilities.^2−5^ These memristors exhibit unique characteristics by colocating both the memory and computing in the same device, storing the information as a modulated resistance. The benefits of this configuration position them as highly efficient artificially intelligent hardware that can mimic the functions of the human brain.^6,7^

Emerging technologies have demonstrated this memristive behavior for more complex and demanding in-memory neuromorphic computation schemes.^8^ These memristive devices range from metal/oxide/metal structures,^9−11^ organic semiconductors,^12,13^ complementary metal/oxide/semiconductor (CMOS) compatible silicon-based devices,^14,15^ and a wide range of metal halide perovskite formulations.^16−19^ Furthermore, specific memristive responses and properties are required depending on the complexity and hardware implementation of the computational schemes and frameworks. These characteristics vary from nonvolatile binary switching for digital in-memory computing and spiking neural networks^6,7,20^ to volatile analog switching for brain-inspired computing and artificial neural networks.^21^ As the field of memristive devices continues to evolve, it is imperative to develop models that provide a deeper understanding of the intricate mechanisms governing their resistive switching behavior.

While the physical mechanisms of the resistive switching and the nature of the internal kinetic processes vary from each material system and configuration, a general formulation for a voltage-controlled memristor is widely accepted, formed by the equations^22−24^
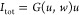
1
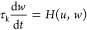
2

The equations relate current *I*_tot_ and voltage *u* in the device via an internal state variable *w*, and *G*(*u*,*w*) is a general conductance function. Equation 2 describes the evolution of the variable *w* in response to the change of external stimuli by the kinetic relaxation time τ_k_ and the general adaptation function *H*(*u*,*w*).

The memristive response of memristors, denoted as *resistive switching*, is defined as the reversible phenomenon of two-terminal elements that changes the resistance upon applying electrical stimuli.^24,25^ Numerous numerical models ranging from space charge limited current (SCLC),^21^ drift-diffusion,^15,26,27^ and SPICE modeling^28−30^ have been proposed to describe the resistive switching of memristors. While being based on the general formulation of eqs 1 and 2, however, the dominant analytical models incorporate a piecewise structure to distinguish the on and off switching cycles, e.g.,^30−34^
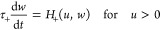
3
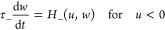
4

This distinction is justified since the current must increase in the set cycle, while it must decrease in the off-switching cycle. However, the separation in voltage (or current^31^) domains produces some computational troubles when different types of stimuli must be probed in separate experiments, as is usual in the analysis of voltage pulses for synaptical properties. In addition, the piecewise approach obscures the underlying physical properties, which should be continuous and differentiable.^35^

In place of the conventional numerical models for semiconductor devices, the memristive response can be approximated using a well-established tool in biology that adopts two main features: an onset of the internal variable *w* at a certain thermodynamic threshold, and a relaxation time τ_k_(*u*) as a unique function of the voltage, across the whole relevant voltage range.^36^ The latter quantity determines the kinetic response.

As an example, the Hodgkin–Huxley (HH) model describes the different conductance responses of ion channel currents (leakage current, delayed-rectified potassium (K^+^) current, and a transient sodium (Na^+^) current) within neurons, responsible for generating the action potentials of neural electrical activity.^37,38^ The HH model incorporates a voltage-controlled activation and deactivation of the channel conductance, but it uses a single voltage-dependent relaxation time for both activation and deactivation of each memory variable. While we will use similar modeling methods, we remark that in this work, we do not address neuron dynamics. We aim to formulate a general dynamical equation for solid-state memristors that can address the response to different types of time-dependent stimuli, including pulsed voltage and resistive switching.

The Letter is organized as follows. First, we show the experimental stable resistive switching of a simple methylammonium bromide (MAPbBr_3_)-based perovskite memristor that will be used to build the model. Further scan rate- and upper vertex voltage dependencies are shown to establish the representative characteristics of these solid-state memristors. Then, we establish a model for the dynamic response of voltage-controlled memristors, adopting a continuous relaxation time inspired by the HH model. The model validation is facilitated by reproducing the characteristic *I*–*V* responses of the representative perovskite-based device, including the scan rate- and upper voltage dependencies. Moreover, the comprehensive validity of the model is demonstrated by simulating the memristive response of a range of memristor material systems and configurations. We show that the model can capture the different device-switching properties ranging from threshold volatile to bipolar nonvolatile memory. Finally, in a direct test of neuromorphic functionalities, we show that the model describes well the response to sequences of voltage pulses that cause synaptic potentiation effects.

The characteristic *I*-*V* curves of the studied perovskite memristor, measured at a scan rate of 1 V s^–1^, in the linear and semilog scales, are shown in Figures 1a and b, respectively, with the inset illustrating the device and measurement configuration. These experimental responses have been previously discussed,^35^ and here, we address a suitable model to explain the kinetic properties. On a linear scale, the device distinctly exhibits a strong inverted hysteresis, typically observed in MAPbBr_3_-based solar cells, indicating an inductive-type time domain response.^39−41^ In the inductive response of a memory device, the current increases with time, and the hysteresis curve traces a counterclockwise loop, as will be further discussed later.^42,43^

**Figure 1:**
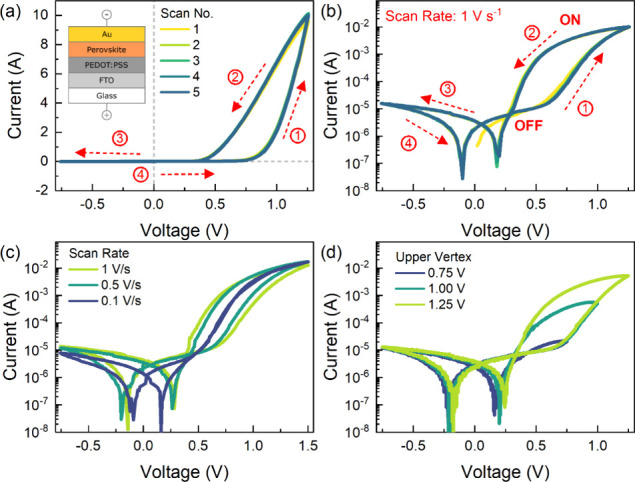
(a) Characteristic *I*–*V* response in the linear scale of the FTO/PEDOT:PSS/MAPbBr_3_/Au memristor device with the inset illustrating the schematic diagram of the device configuration, (b) characteristic *I*–*V* response represented in the semilog scale with the arrows and numbers indicating the scan direction, (c) scan rate-dependent *I*–*V* curves with the reconstructed *I*–*V* of the impedance measurements, and (d) upper vertex-dependent multilevel/analog resistive switching of the memristor device.

In the semilog scale, the perovskite memristor features a threshold resistive switching in the positive polarity with an ON/OFF ratio of ∼2 orders of magnitude.^44−46^ At low voltages, the memristor is at its initial high resistance state (HRS), also denoted as the OFF state. In Figure 1a,b, the activation process in positive voltages is indicated as (1) in the forward direction and deactivation as (2) in the reverse direction. As the forward voltage scan approaches positive voltage and passes beyond the threshold voltage of ∼0.6 V, the device gradually transitions from the HRS to the low resistance state (LRS), also denoted as the ON state in binary switching, promoting the SET process.^47^ Notably, in the reverse scan direction, the ON state is maintained until the voltage reaches a lower threshold voltage of ∼0.3 V, where the device transitions from the LRS back to the HRS (RESET process). This characteristic resistive switching exhibits volatile memory where the ON state relaxes back to the OFF state upon sufficiently reduced applied voltage.^18,48,49^ The scan rate-dependent characteristic *I*–*V* curves (Figure 1c) further demonstrate the inductive response of the perovskite memristor, which exhibits a decreasing forward scan current with an increasing scan rate.^50,51^ Moreover, by varying the upper vertex of the *I*–*V* measurements (Figure 1d), the device displays a multilevel/multistate resistive switching suitable for analog volatile memory applications, reproducing short-term memory (STM) behavior in neuromorphic systems.^7,52^

Based on the general framework of eqs 1 and 2, and the observed experimental features, we now describe a set of characteristics that the model must incorporate. In the memristors used for neuromorphic computation,^53^ the system transitions from a low conducting state (of conductance *g*_L_) HRS, to a high conductance state of conductance *g*_H_, LRS, where *g*_H_ ≫ *g*_L_. When the voltage applied is *u*, we have a basic current *I*_tot_ = *g*_L_*u*, which increases when the resistance switches. We establish the control of conductance by a memory variable 0 ≤ *X* ≤ 1 as follows.
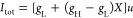
5

The equilibrium value of the memory variable *X*_ss_ is a sigmoidal activation function^32,54,55^
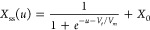
6

The threshold voltage *V*_t_ defines and translates the state transition of *X*_ss_ from its initial OFF state, *X*_ss_ = *X*_0_, to its ON state, *X*_ss_ ≈ 1 (Figure 2a). On the other hand, *V*_m_ controls the steepness and voltage window of the state transition, where a low *V*_m_ exhibits an abrupt state transition at a narrow voltage window, while a high *V*_m_ indicates a gradual state transition at a wider voltage window (Figure 2b).

**Figure 2:**
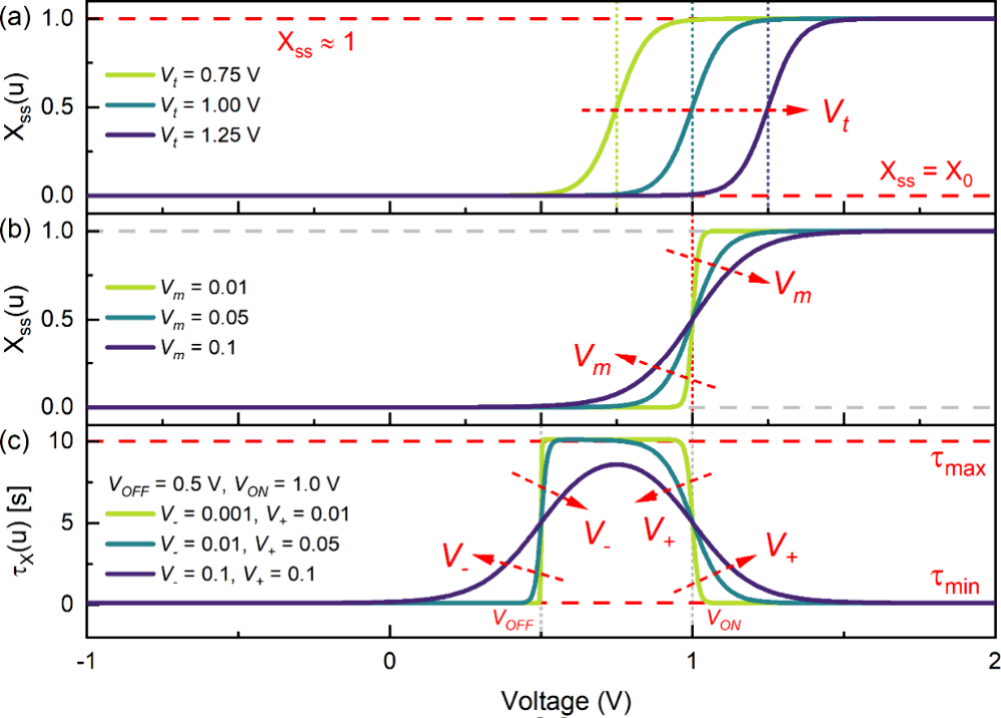
Voltage-dependent internal state variable function *X*_ss_(*u*) of the dynamical neuron-based model with varying (a) voltage parameter *V*_t_ and (b) ideality factor parameter *V*_m_. Voltage-dependent time constant function τ_*X*_(*u*) at fixed voltage parameters *V*_OFF_ = 0.5 V and *V*_ON_ = 1.0 V with varying ideality factor parameters *V*_–_ and *V*_+_.

We have mentioned the dominant feature of the resistance switching between two conducting states. As demonstrated in the experimental response of the perovskite-based memristor, this threshold resistive switching from the OFF state to the ON state can relax back to the OFF state with the removal or sufficient reduction of the applied electrical stimulus, indicating a volatile memory. In contrast, another typical resistive switching behavior can maintain both the OFF and ON states for longer durations (up to 10 years) upon removing the voltage stimulus, indicating a nonvolatile memory.^21^ These two main types of resistive switching behaviors are considered in formulating our conductance-based memristor model, as discussed in more detail in the following.

The first important point of the model is the need for the high conductance state to be activated at a SET potential and deactivated at a certain RESET potential. Therefore, the *X* = 1 state must decay back to *X* = *X*_0_ to reverse the initial switching. This very basic feature is well acknowledged in solid-state memristor models, yet it is normally achieved by piecewise splitting of the *X* dynamics into different equations for different voltage domains, as indicated in eqs 3 and 4.^30−33^ This approach is unsatisfactory as it hides the kinetic origin of the memristor RESET process.

The central idea of the model, in analogy to biological ion models, is to precisely formulate the kinetic form in which the activation time can be modulated by the voltage, apart from the stationary characteristic of eq 6. The kinetic response of halide perovskite memristors has been investigated, and the different mechanisms have been recently reviewed.^56^ The switching dynamics is associated with ionic filament formation of interface barrier modification. More generally, in solid-state memristors, the electrochemical processes for filament formation, involving nucleation, charge transfer, and ion transport, produce a nonlinear exponential voltage–time relationship.^57^ It has been reported that relaxation time is a strong function of the voltage in two-terminal resistance switches and analog memristors based on ionic motion,^58−62^ as well as in biological^37,63−65^ and artificial fluidic channels.^66^

Based on these general properties, it is reasonable to adopt a double exponential form of τ_*X*_(*u*), that will decrease both at very positive and very negative voltages with respect to the thermodynamic activation voltage *V*_t_. The deactivation of the solid-state memristor at reverse voltage will occur when *u* < *V*_t_, switching back to *X* = 0. However, if τ_k_ is long enough, the deactivation is kinetically impeded. As a result, we propose that the kinetic time vanishes at a RESET threshold voltage *V*_OFF_, more negative than the equilibrium threshold voltage *V*_t_. This feature will determine the memristor’s volatility at *V*_OFF_ ≈ *V*_t_, or if it holds the high conductance below the thermodynamic threshold, when *V*_OFF_ ≪ *V*_t_.

Similarly, the kinetical changes may disappear at high voltage if *X* is already in the ON state, when *X* = 1, since *u* > *V*_t_, without additional kinetic changes. Again, here we have τ_k_ → 0 after some SET threshold voltage *V*_ON_, where we observe experimentally a hysteresis free region of the *I*–*V* curve at voltages higher than that value. This is due to the lack of memory effect once τ_k_ ≈ 0. The hysteresis-free region above the transition to the high conductive state can be displayed as a linear increase of current with voltage, which is discussed further later, and several experimental examples will be shown.

Combining these physical features, we obtain the following model defined by
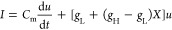
7
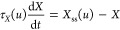
8where
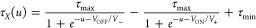
9Here, *C*_m_ is the device capacitance. The evolution of *X* is dependent on the kinetic time constant function τ_*X*_(*u*) that is a specific form of τ_*k*_ of eq 2. The voltage-dependence of τ_*X*_(*u*) is defined by the characteristic maximum kinetic time constant τ_*max*_, the minimum kinetic time constant τ_min_, the threshold RESET voltage *V*_OFF_ with its corresponding ideality factor *V*_–_, and the threshold SET voltage *V*_ON_ with its corresponding ideality factor *V*_+_.

The voltage-dependent forms of the *X*_ss_(*u*) with varying *V*_t_ and *V*_m_, and τ_*X*_(*u*) is illustrated in Figure 2. The threshold SET and RESET voltages and their corresponding ideality factor parameters affect the τ_*X*_(*u*) in the same manner as shown in Figure 2c. The *V*_OFF_ defines the threshold voltage, where τ_*X*_(*u*) transitions from τ_*X*_ = τ_min_ to , with *V*_–_ controlling the steepness of the transition. On the other hand, *V*_ON_ defines the threshold voltage where τ_*X*_ reversibly transitions back from τ_max_ to τ_min_, with *V*_+_ controlling the steepness of transition. Notably, when the threshold voltages are close enough with gradual and wide voltage windows, both transitions can overlap, and the time constant begins to decrease back to τ_min_ before reaching the τ_max_. In this work, we do not provide a further microscopic investigation into the origin of eq 9, but we will show that this form explains diverse types of characteristic memristor dynamic responses.

In summary, eqs 7–9 form the model we use hereafter. The distinctive property of this model is that both constitutive eqs 7 and 8 are linear in the *X* variable, while the nonlinearity is restricted to the  functions. As the model (eqs 7–9) is based on the kinetic control of the normalized conductance *X*, we denominate this a conductance-activated quasi-linear memristor (CALM) model. We implement this model to reproduce the experimental results obtained from the perovskite-based memristor presented in Figure 1.

From the characteristic functions of the model, the parameters are selected to match the experimental trends with the simulated *I*-*V* curves shown in Figure 3, as listed in Table S1. Notably, the model can replicate the inductive hysteresis in the linear and log scales (Figure 3a,b) and the threshold switching with an ON/OFF ratio of ∼2 orders of magnitude at a scan rate of 1 V s^–1^. This volatile threshold switching is obtained by having *V*_ON_ = 1.05 V and *V*_OFF_ = 0.75 V both in the positive polarity, while the ON/OFF ratio is controlled by the proper selection of the conductivities *g*_L_ and *g*_H_. In addition, using the same parameters and only varying the scan rate d*u*/d*t*, the simulated scan rate-dependent characteristic *I*–*V* device response is highly consistent with the experimental response exhibiting a decreasing hysteresis with the reduction of scan rate (Figure 3c).

**Figure 3:**
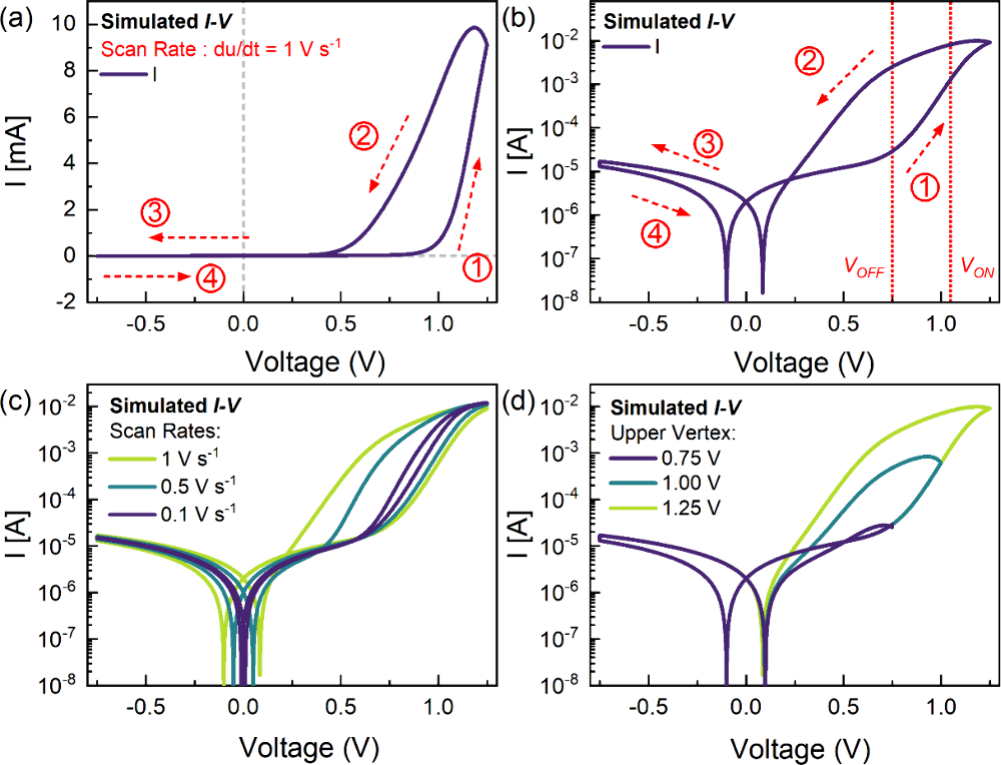
Simulated characteristic *I*–*V* response in the (a) linear and (b) semilog scales with the arrows and numbers indicating the scan direction and sequence, (c) simulated scan rate-dependent *I*–*V* response, and (d) simulated upper vertex-dependent multilevel/analog resistive switching using the dynamical neuron-based model.

Lastly, by changing the upper vertex voltage of the simulated *I*–*V* scans, the analog multilevel/multistate resistive switching is obtained in agreement with the actual response of the memristor (Figure 3d). The corresponding voltage-dependent curves of the pertinent parameters *X*_ss_(*u*), τ_*X*_(*u*), and the dynamic evolution of *X*(*u*) are shown in Figure S1. These results demonstrate the CALM model’s capacity to reproduce the characteristic *I*–*V* response of volatile perovskite-based memristors, including key aspects such as ON-OFF ratio variation, scan rate dependence, and upper vertex voltage-dependent multistate analog switching.

Going a step further, we aim to prove that the CALM model can reproduce the behavior of both volatile and nonvolatile memristors by precisely selecting the characteristic parameters of the kinetic time constant function τ_*X*_(*u*). As previously discussed, modifying the value of the threshold RESET voltage *V*_OFF_ and increasing the LRS to HRS transition time can impede the deactivation kinetics. Figure 4a-i summarizes experimental characteristics of three different perovskite memristor configurations exhibiting varying types of memory and their corresponding simulated responses by controlling the threshold voltages *V*_OFF_ and *V*_ON_, and their respective ideality factor parameters *V*_–_ and *V*_+_. The parameter list of the simulated *I*–*V* curves are displayed in Table S2. For the FTO/PEDOT:PSS/MAPbBr_3_/Au device exhibiting threshold volatile switching (Figure 4a), both *V*_OFF_ and *V*_ON_ are in the positive polarity with relatively steep but gradual *V*_–_ and *V*_+_ (Figure 4d). With these characteristic parameters, τ_*X*_(*u*) transitions from τ_min_ to τ_max_ and back to τ_min_ only in the positive voltages (Figure 4g). Hence, with the removal or sufficient reduction of the applied voltage, the memristor devices relax back to the OFF state, indicating volatile memory.^44^

**Figure 4:**
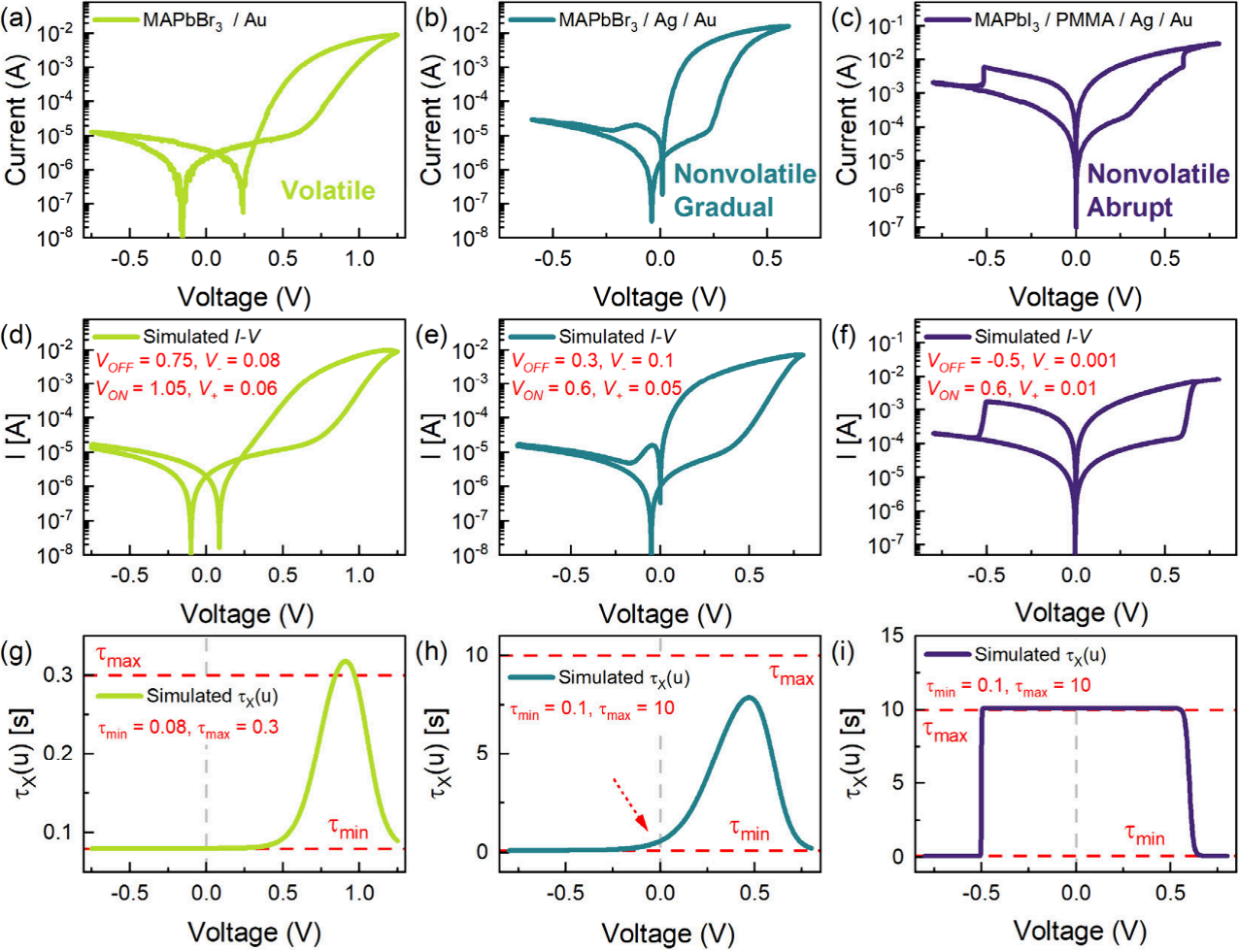
Experimental characteristic *I*–*V* response in the semilog scale of a (a) FTO/PEDOT:PSS/MAPbBr_3_/Au memristor exhibiting volatile memory, a (b) FTO/MAPbBr_3_/Ag/Au memristor exhibiting nonvolatile memory with gradual SET and RESET, and a (c) FTO/PEDOT:PSS/MAPbI_3_/PMMA/Ag/Au memristor^45^ exhibiting nonvolatile memory with abrupt SET and RESET. The corresponding simulated *I*–*V* responses using the dynamical neuron-based model reproducing the different memory variations: (d) volatile, (e) nonvolatile with gradual SET and RESET, and (f) nonvolatile with abrupt SET and RESET with pertinent parameters indicated. (g–i) Associated relaxation time functions.

In contrast, for the FTO/PEDOT:PSS/MAPbI_3_/PMMA/Ag/Au exhibiting nonvolatile abrupt binary switching^45^ (Figure 4c), *V*_OFF_ is negative, while *V*_ON_ is positive with highly abrupt *V*_–_ and *V*_+_ values (Figure 4f–i). These parameters indicate that τ_*X*_(*u*) abruptly transitions to τ_max_ in the negative polarity and again abruptly transitions back to τ_min_ in the positive polarity. This implies that a negative applied voltage is required to promote the RESET process, and the device will stay in the ON state upon the removal of the applied voltage, indicating a nonvolatile memory.^44^ In between these two extreme cases, an FTO/PEDOT:PSS/MAPbBr_3_/Ag/Au device exhibits a nonvolatile but gradual resistive switching (Figure 4b). For this memristor, both *V*_OFF_ and *V*_ON_ are positive, similar to the volatile case, however, *V*_–_ is highly gradual with a wider transition voltage window (Figure 4e). The resulting τ_*X*_(*u*) begins to transition to τ_max_ at negative voltages near zero up to positive voltages then transitions back to τ_min_ without reaching τ_max_ (Figure 4h). This suggests that a certain negative voltage is required to fully RESET the device, but the removal of applied voltage would retain the ON state for a specific time defined by the relaxation constant of the kinetic process.

At this point, we have demonstrated that the CALM model is able to reproduce the volatile, nonvolatile gradual, and nonvolatile abrupt switching of perovskite-based memristors with the proper selection of the characteristic parameters of the kinetic time constant function τ_*X*_(*u*). We can extend the implementation of the model to reproduce some of the reported characteristic *I*-*V* responses from memristor devices of various material systems and configurations, including W/SiGe/a-Si/Ag, Si/SiO_2_/Ag and SrRuO_3_/Cr-SrZrO_3_/Au memristors. In Figure S2, we address various oxide-based memristors with Ag ions, exhibiting volatile threshold switching for analog applications in bioinspired synaptic functions. In Figure S3 we apply the model to the analysis of memristors with bipolar nonvolatile resistive switching for binary in-memory applications.

The wide range of memristive responses that our CALM model has been able to consistently reproduce paves the way for a more holistic and systematic approach in the memristor properties by the analysis of the characteristic *I*–*V* curves. However, *X*-linearity it is not a universal property of memristors, which can contain high nonlinearity in both *G*,*H* functions of eqs 1 and 2. The CALM model, in its current form, cannot emulate certain complex resistive switching situations.^67,68^ In complementary resistive switching, it is necessary to add two memristors in antiserial configuration.^69,70^ Other memristors require more than one memory variable.^46,71^ A generalization of the model would require the addition of several coupled state variables and series/parallel combinations.^72^

The analysis of current–voltage curves at varying scan rates serves as a valuable tool for characterizing the behavior of memristors and provides crucial insights into their switching properties. However, it is essential to recognize that the operational dynamics of memristors significantly differ when employed in practical applications. In real-world scenarios, memristors respond to sequences of voltage pulses with distinct characteristics, undergoing changes in their conductive state based on pulse attributes such as applied voltage or pulse width. With appropriate stimulation, memristors must gradually transition to the ON state, exhibiting increased conductivity and current potentiation.

The synaptic behavior of halide perovskite memristors was discussed recently.^35^ An illustrative example of such potentiation is presented in Figure S4, showcasing the experimental response of a halide perovskite memristor to various voltage pulse trains. The observation reveals that current potentiation does not manifest for low voltages, while it becomes evident at higher voltage levels. Figure 5 demonstrates that the CALM model can replicate these results. In Figure 5a we illustrate how the current achieved in each successive voltage pulse surpasses that of the preceding one, indicating a progressive increase in conductivity. Figure 5b shows the transients in more detail.

**Figure 5:**
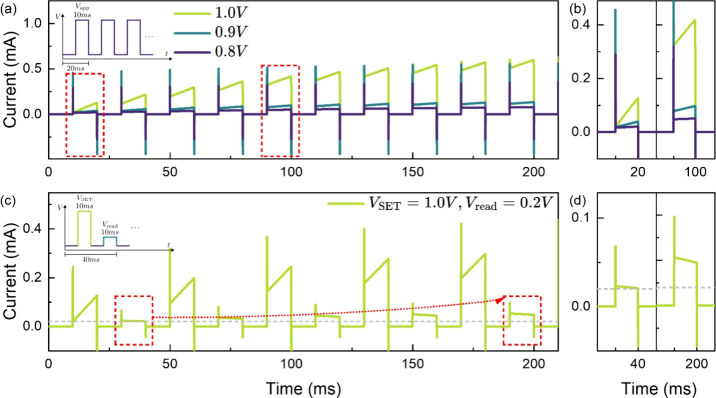
Simulated (a) voltage-dependent transient current response of the FTO/PEDOT:PSS/MAPbBr_3_/Au memristor (model parameters in Table S1) using a voltage train stimulus of 10 identical pulses with a pulse width of 10 ms and a pulse period of 20 ms at varying applied voltages potentiation (schematic diagram shown in the inset) exhibiting synaptic, (b) the magnified view of the transient response of the first voltage pulse. (c) The simulated response of the current to a train of 1.0 V steps, followed by a read voltage of 0.2 V where the evolution of the current can be distinguished, and (d) the magnified view of the first and the last reading signals.

We further analyze the model’s response to typical sequences of SET and read voltages, which are crucial for neuromorphic applications. These sequences comprise a series of voltage steps at an activation voltage *V*_SET_, followed by a read voltage *V*_read_, enabling the monitoring of the conductive state through current measurements at each step. As illustrated in Figure 5c,d, the current increases progressively at each read step, consistent with the application of short SET pulses that incrementally enhance the conductive state. The magnified view in Figure 5d highlights how the conductive state doubles within just five brief pulses of a few milliseconds each. This outcome underscores the model’s effectiveness in reproducing the current–voltage characteristics of memristors and capturing the current potentiation essential for the device’s operation in practical applications and synaptic systems. Notably, the current evolution during these read sequences reveals the dynamics of the model’s main variable, *X*, which dictates the devices’ conductive state. Understanding and predicting the behavior of the state variable *X* is essential for elucidating memristors operations and, making the CALM model a valuable tool for exploring their functionality.

In conclusion, we have formulated a dynamical model of the characteristic *I*–*V* response of memristors by incorporating internal state functions and the voltage-dependent kinetic time function (relaxation time) that governs their time response. The CALM model, inspired in biological channel models, adopts linearity of the internal variable *X* and emulates the volatile and nonvolatile resistive switching in various device systems and configurations. The model reproduces the experimental characteristics of scan rate-dependent hysteresis and the analog multilevel/multistate resistive switching of a volatile perovskite-based memristor. The versatility of this method is showcased through its capability to simulate different types of memristors by systematically adjusting parameters related to the characteristic kinetic time constant function. A simple description of the memristors is obtained with only the necessary number of parameters to describe the electrical response, that reproduces the most common types of resistive switching. As memristors are intended to operate as neuromorphic devices, a description of the memristive response based on a smooth and continuous CALM model provides a more integrated approach in device characterization and application. This model will serve as a crucial tool for investigating regular memristors and as a starting point for more complicated memristive behaviors. As the research landscape evolves, this work contributes to memristors’ ongoing application and design, emphasizing their potential impact on the future of electronics and computing.

## Supporting Information Available

Experimental; Simulation details; Transient current response (PDF)

## References

[ref1] Christensen D. V.; Dittmann R.; Linares-Barranco B.; Sebastian A.; Le Gallo M.; Redaelli A.; Slesazeck S.; Mikolajick T.; Spiga S.; Menzel S.; Valov I.; Milano G.; Ricciardi C.; Liang S.-J.; Miao F.; Lanza M.; Quill T. J.; Keene S. T.; Salleo A.; Grollier J.; Marković D.; Mizrahi A.; Yao P.; Yang J. J.; Indiveri G.; Strachan J. P.; Datta S.; Vianello E.; Valentian A.; Feldmann J.; Li X.; Pernice W. H. P.; Bhaskaran H.; Furber S.; Neftci E.; Scherr F.; Maass W.; Ramaswamy S.; Tapson J.; Panda P.; Kim Y.; Tanaka G.; Thorpe S.; Bartolozzi C.; Cleland T. A.; Posch C.; Liu S.; Panuccio G.; Mahmud M.; Mazumder A. N.; Hosseini M.; Mohsenin T.; Donati E.; Tolu S.; Galeazzi R.; Christensen M. E.; Holm S.; Ielmini D.; Pryds N. 2022 roadmap on neuromorphic computing and engineering. Neuromorphic Computing and Engineering 2022, 2, 022501 10.1088/2634-4386/ac4a83.

[ref2] Kim K.-H.; Gaba S.; Wheeler D.; Cruz-Albrecht J. M.; Hussain T.; Srinivasa N.; Lu W. A Functional Hybrid Memristor Crossbar-Array/CMOS System for Data Storage and Neuromorphic Applications. Nano Lett. 2012, 12, 389–395. 10.1021/nl203687n. 22141918

[ref3] Wang Z.; Joshi S.; Savel’ev S. E.; Jiang H.; Midya R.; Lin P.; Hu M.; Ge N.; Strachan J. P.; Li Z.; Wu Q.; Barnell M.; Li G.-L.; Xin H. L.; Williams R. S.; Xia Q.; Yang J. J. Memristors with diffusive dynamics as synaptic emulators for neuromorphic computing. Nat. Mater. 2017, 16, 101–108. 10.1038/nmat4756. 27669052

[ref4] Ilyas N.; Li C.; Wang J.; Jiang X.; Fu H.; Liu F.; Gu D.; Jiang Y.; Li W. A Modified SiO2-Based Memristor with Reliable Switching and Multifunctional Synaptic Behaviors. J. Phys. Chem. Lett. 2022, 13, 884–893. 10.1021/acs.jpclett.1c03912. 35049317

[ref5] Lanza M.; Sebastian A.; Lu W. D.; Le Gallo M.; Chang M.-F.; Akinwande D.; Puglisi F. M.; Alshareef H. N.; Liu M.; Roldan J. B. Memristive technologies for data storage, computation, encryption, and radio-frequency communication. Science 2022, 376, eabj9979 10.1126/science.abj9979. 35653464

[ref6] Ielmini D.; Wong H. S. P. In-memory computing with resistive switching devices. Nature Electronics 2018, 1, 333–343. 10.1038/s41928-018-0092-2.

[ref7] Mannocci P.; Farronato M.; Lepri N.; Cattaneo L.; Glukhov A.; Sun Z.; Ielmini D. In-memory computing with emerging memory devices: Status and outlook. APL Machine Learning 2023, 1, 010902 10.1063/5.0136403.

[ref8] Ielmini D.; Wang Z.; Liu Y. Brain-inspired computing via memory device physics. APL Materials 2021, 9, 050702 10.1063/5.0047641.

[ref9] Lee A. R.; Bae Y. C.; Baek G. H.; Chung J. B.; Lee S. H.; Im H. S.; Hong J. P. Multifunctional resistive switching behaviors employing various electroforming steps. Journal of Materials Chemistry C 2016, 4, 823–830. 10.1039/C5TC03303A.

[ref10] Strukov D. B.; Snider G. S.; Stewart D. R.; Williams R. S. The missing memristor found. Nature 2008, 453, 80–83. 10.1038/nature06932. 18451858

[ref11] Huang H. M.; Yang R.; Tan Z. H.; He H. K.; Zhou W.; Xiong J.; Guo X. Quasi-Hodgkin–Huxley Neurons with Leaky Integrate-and-Fire Functions Physically Realized with Memristive Devices. Adv. Mater. 2019, 31, 1803849–1803849. 10.1002/adma.201803849. 30461092

[ref12] Melianas A.; Quill T. J.; LeCroy G.; Tuchman Y.; Loo H. V.; Keene S. T.; Giovannitti A.; Lee H. R.; Maria I. P.; McCulloch I.; Salleo A. Temperature-resilient solid-state organic artificial synapses for neuromorphic computing. Science Advances 2020, 6, 1–7. 10.1126/sciadv.abb2958. PMC7458436 32937458

[ref13] Van De Burgt Y.; Lubberman E.; Fuller E. J.; Keene S. T.; Faria G. C.; Agarwal S.; Marinella M. J.; Alec Talin A.; Salleo A. A non-volatile organic electrochemical device as a low-voltage artificial synapse for neuromorphic computing. Nat. Mater. 2017, 16, 414–418. 10.1038/nmat4856. 28218920

[ref14] Ilyas N.; Li C.; Wang J.; Jiang X.; Fu H.; Liu F.; Gu D.; Jiang Y.; Li W. A Modified SiO2-Based Memristor with Reliable Switching and Multifunctional Synaptic Behaviors. J. Phys. Chem. Lett. 2022, 13, 884–893. 10.1021/acs.jpclett.1c03912. 35049317

[ref15] Wang W.; Covi E.; Lin Y. H.; Ambrosi E.; Milozzi A.; Sbandati C.; Farronato M.; Ielmini D. Switching Dynamics of Ag-Based Filamentary Volatile Resistive Switching Devices - Part II: Mechanism and Modeling. IEEE Trans. Electron Devices 2021, 68, 4342–4349. 10.1109/TED.2021.3095033.

[ref16] Beom K.; Fan Z.; Li D.; Newman N. Halide perovskite based synaptic devices for neuromorphic systems. Materials Today Physics 2022, 24, 100667–100667. 10.1016/j.mtphys.2022.100667.

[ref17] Xiao Z.; Huang J. Energy-Efficient Hybrid Perovskite Memristors and Synaptic Devices. Advanced Electronic Materials 2016, 2, 1–8. 10.1002/aelm.201600100.

[ref18] John R. A.; Demirağ Y.; Shynkarenko Y.; Berezovska Y.; Ohannessian N.; Payvand M.; Zeng P.; Bodnarchuk M. I.; Krumeich F.; Kara G.; Shorubalko I.; Nair M. V.; Cooke G. A.; Lippert T.; Indiveri G.; Kovalenko M. V. Reconfigurable halide perovskite nanocrystal memristors for neuromorphic computing. Nat. Commun. 2022, 13, 1–10. 10.1038/s41467-022-29727-1. 35440122 PMC9018677

[ref19] Sakhatskyi K.; John R. A.; Guerrero A.; Tsarev S.; Sabisch S.; Das T.; Matt G. J.; Yakunin S.; Cherniukh I.; Kotyrba M.; Berezovska Y.; Bodnarchuk M. I.; Chakraborty S.; Bisquert J.; Kovalenko M. V. Assessing the Drawbacks and Benefits of Ion Migration in Lead Halide Perovskites. ACS Energy Lett. 2022, 7, 3401–3414. 10.1021/acsenergylett.2c01663. 36277137 PMC9578653

[ref20] Zidan M. A.; Strachan J. P.; Lu W. D. The future of electronics based on memristive systems. Nature Electronics 2018, 1, 22–29. 10.1038/s41928-017-0006-8.

[ref21] Zhu J.; Zhang T.; Yang Y.; Huang R. A comprehensive review on emerging artificial neuromorphic devices. Appl. Phys. Rev. 2020, 7, 011312 10.1063/1.5118217.

[ref22] Chua L. O.; Kang S. M. Memristive devices and systems. Proceedings of the IEEE 1976, 64, 209–223. 10.1109/PROC.1976.10092.

[ref23] Bisquert J. Current-controlled memristors: resistive switching systems with negative capacitance and inverted hysteresis. Phys. Rev. Appl. 2023, 20, 044022 10.1103/PhysRevApplied.20.044022.

[ref24] Pershin Y. V.; Di Ventra M. Memory effects in complex materials and nanoscale systems. Adv. Phys. 2011, 60, 145–227. 10.1080/00018732.2010.544961.

[ref25] Di Ventra M.; Pershin Y. V. On the physical properties of memristive, memcapacitive and meminductive systems. Nanotechnology 2013, 24, 255201–255201. 10.1088/0957-4484/24/25/255201. 23708238

[ref26] Strukov D. B.; Borghetti J. L.; Williams R. S. Coupled ionic and electronic transport model of thin-film semiconductor memristive behavior. Small 2009, 5, 1058–1063. 10.1002/smll.200801323. 19226597

[ref27] Strukov D. B.; Williams R. S. Exponential ionic drift: Fast switching and low volatility of thin-film memristors. Applied Physics A: Materials Science and Processing 2009, 94, 515–519. 10.1007/s00339-008-4975-3.

[ref28] Fang X.; Yang X.; Wu J.; Yi X. A compact SPICE model of unipolar memristive devices. IEEE Transactions on Nanotechnology 2013, 12, 843–850. 10.1109/TNANO.2013.2275178.

[ref29] Khadar Basha N.; Ramashri T. Spice model of Memristor with threshold switching characteristics. ARPN Journal of Engineering and Applied Sciences 2018, 13, 2581–2587.

[ref30] Kvatinsky S.; Friedman E. G.; Kolodny A.; Weiser U. C. TEAM: Threshold adaptive memristor model. IEEE Transactions on Circuits and Systems I: Regular Papers 2013, 60, 211–221. 10.1109/TCSI.2012.2215714.

[ref31] Pickett M. D.; Strukov D. B.; Borghetti J. L.; Yang J. J.; Snider G. S.; Stewart D. R.; Williams R. S. Switching dynamics in titanium dioxide memristive devices. J. Appl. Phys. 2009, 106, 074508 10.1063/1.3236506.

[ref32] Miranda E.; Suñé J. Memristive State Equation for Bipolar Resistive Switching Devices Based on a Dynamic Balance Model and Its Equivalent Circuit Representation. IEEE Transactions on Nanotechnology 2020, 19, 837–840. 10.1109/TNANO.2020.3039391.

[ref33] Gao L.; Ren Q.; Sun J.; Han S.-T.; Zhou Y. Memristor modeling: challenges in theories, simulations, and device variability. J. Mater. Chem. C 2021, 9, 16859–16884. 10.1039/D1TC04201G.

[ref34] Miranda E.; Piros E.; Aguirre F. L.; Kim T.; Schreyer P.; Gehrunger J.; Oster T.; Hofmann K.; Suñé J.; Hochberger C.; Alff L. Simulation of Bipolar-Type Resistive Switching Devices Using a Recursive Approach to the Dynamic Memdiode Model. IEEE Electron Device Lett. 2023, 44, 1551–1554. 10.1109/LED.2023.3298023.

[ref35] Gonzales C.; Bou A.; Guerrero A.; Bisquert J. Capacitive and Inductive Characteristics of Volatile Perovskite Resistive Switching Devices with Analog Memory. J. Phys. Chem. Lett. 2024, 15, 6496–6503. 10.1021/acs.jpclett.4c00945. 38869927 PMC11215770

[ref36] Bisquert J. Hysteresis, Rectification and Relaxation Times of Nanofluidic Pores for Neuromorphic Circuit Applications. Advanced Physics Research 2024, 3, 2400029 10.1002/apxr.202400029.

[ref37] Hodgkin A. L.; Huxley A. F. A quantitative description of membrane current and its application to conduction and excitation in nerve. J. Physiol 1952, 117, 500–544. 10.1113/jphysiol.1952.sp004764. 12991237 PMC1392413

[ref38] Dayan P.; Abbott L. F. Theoretical Nueroscience - Computational and Mathematical Modeling of Neural Systems; MIT Press: London, 2001.

[ref39] Bisquert J.; Guerrero A.; Gonzales C. Theory of Hysteresis in Halide Perovskites by Integration of the Equivalent Circuit. ACS Physical Chemistry Au 2021, 1, 25–44. 10.1021/acsphyschemau.1c00009. 36855663 PMC9718316

[ref40] Fabregat-Santiago F.; Kulbak M.; Zohar A.; Vallés-Pelarda M.; Hodes G.; Cahen D.; Mora-Seró I. Deleterious Effect of Negative Capacitance on the Performance of Halide Perovskite Solar Cells. ACS Energy Lett. 2017, 2, 2007–2013. 10.1021/acsenergylett.7b00542.

[ref41] Gonzales C.; Guerrero A.; Bisquert J. Transition from Capacitive to Inductive Hysteresis: A Neuron-Style Model to Correlate I- V Curves to Impedances of Metal Halide Perovskites. J. Phys. Chem. C 2022, 126, 13560–13578. 10.1021/acs.jpcc.2c02729.

[ref42] Bisquert J.; Guerrero A. Chemical Inductor. J. Am. Chem. Soc. 2022, 144, 5996–6009. 10.1021/jacs.2c00777. 35316040 PMC8991013

[ref43] Bisquert J. Inductive and capacitive hysteresis of current-voltage curves. A unified structural dynamics in solar energy devices, memristors, ionic transistors and bioelectronics. PRX Energy 2024, 3, 011001 10.1103/PRXEnergy.3.011001.

[ref44] Lanza M.; Wong H. S. P.; Pop E.; Ielmini D.; Strukov D.; Regan B. C.; Larcher L.; Villena M. A.; Yang J. J.; Goux L.; Belmonte A.; Yang Y.; Puglisi F. M.; Kang J.; Magyari-Köpe B.; Yalon E.; Kenyon A.; Buckwell M.; Mehonic A.; Shluger A.; Li H.; Hou T. H.; Hudec B.; Akinwande D.; Ge R.; Ambrogio S.; Roldan J. B.; Miranda E.; Suñe J.; Pey K. L.; Wu X.; Raghavan N.; Wu E.; Lu W. D.; Navarro G.; Zhang W.; Wu H.; Li R.; Holleitner A.; Wurstbauer U.; Lemme M. C.; Liu M.; Long S.; Liu Q.; Lv H.; Padovani A.; Pavan P.; Valov I.; Jing X.; Han T.; Zhu K.; Chen S.; Hui F.; Shi Y. Recommended Methods to Study Resistive Switching Devices. Advanced Electronic Materials 2019, 5, 1–28. 10.1002/aelm.201800143.

[ref45] Gonzales C.; Guerrero A. Mechanistic and Kinetic Analysis of Perovskite Memristors with Buffer Layers: The Case of a Two-Step Set Process. J. Phys. Chem. Lett. 2023, 14, 1395–1402. 10.1021/acs.jpclett.2c03669. 36738280 PMC9940207

[ref46] Berruet M.; Pérez-Martínez J. C.; Romero B.; Gonzales C.; Al-Mayouf A. M.; Guerrero A.; Bisquert J. Physical Model for the Current–Voltage Hysteresis and Impedance of Halide Perovskite Memristors. ACS Energy Letters 2022, 7, 1214–1222. 10.1021/acsenergylett.2c00121.

[ref47] Gogoi H. J.; Bajpai K.; Mallajosyula A. T.; Solanki A. Advances in Flexible Memristors with Hybrid Perovskites. J. Phys. Chem. Lett. 2021, 12, 8798–8825. 10.1021/acs.jpclett.1c02105. 34491743

[ref48] Pérez-Martínez J. C.; Berruet M.; Gonzales C.; Salehpour S.; Bahari A.; Arredondo B.; Guerrero A. Role of Metal Contacts on Halide Perovskite Memristors. Adv. Funct. Mater. 2023, 33 (47), 202305211 10.1002/adfm.202305211.

[ref49] Lin J.; Ye W.; Zhang X.; Lian Q.; Wu S.; Guo T.; Chen H. A Memristor-Based Leaky Integrate-and-Fire Artificial Neuron With Tunable Performance. IEEE Electron Device Lett. 2022, 43, 1231–1234. 10.1109/LED.2022.3184671.

[ref50] Alvarez A. O.; Arcas R.; Aranda C. A.; Bethencourt L.; Mas-Marzá E.; Saliba M.; Fabregat-Santiago F. Negative Capacitance and Inverted Hysteresis: Matching Features in Perovskite Solar Cells. J. Phys. Chem. Lett. 2020, 11, 8417–8423. 10.1021/acs.jpclett.0c02331. 32903005

[ref51] Shen H.; Jacobs D. A.; Wu Y.; Duong T.; Peng J.; Wen X.; Fu X.; Karuturi S. K.; White T. P.; Weber K.; Catchpole K. R. Inverted Hysteresis in CH 3 NH 3 PbI 3 Solar Cells: Role of Stoichiometry and Band Alignment. J. Phys. Chem. Lett. 2017, 8, 2672–2680. 10.1021/acs.jpclett.7b00571. 28557465

[ref52] Kim S. Y.; Park D. A.; Park N. G. Synthetic Powder-Based Thin (<0.1 μm) Cs3Bi2Br9Perovskite Films for Air-Stable and Viable Resistive Switching Memory. ACS Applied Electronic Materials 2022, 4, 2388–2395. 10.1021/acsaelm.2c00201.

[ref53] Waser R.; Aono M. Nanoionics-based resistive switching memories. Nat. Mater. 2007, 6, 833–840. 10.1038/nmat2023. 17972938

[ref54] Bisquert J.; Bou A.; Guerrero A.; Hernández-Balaguera E. Resistance transient dynamics in switchable perovskite memristors. APL Machine Learning 2023, 1, 036101 10.1063/5.0153289.

[ref55] Munoz-Diaz L.; Rosa A. J.; Bou A.; Sanchez R. S.; Romero B.; John R. A.; Kovalenko M. V.; Guerrero A.; Bisquert J. Inductive and Capacitive Hysteresis of Halide Perovskite Solar Cells and Memristors Under Illumination. Frontiers in Energy Research 2022, 10, 914115 10.3389/fenrg.2022.914115.

[ref56] Kim S.-Y.; Zhang H.; Rubio-Magnieto J. Operating Mechanism Principles and Advancements for Halide Perovskite-Based Memristors and Neuromorphic Devices. J. Phys. Chem. Lett. 2024, 15, 10087–10103. 10.1021/acs.jpclett.4c02170. 39331473 PMC11472375

[ref57] Menzel S.; Tappertzhofen S.; Waser R.; Valov I. Switching kinetics of electrochemical metallization memory cells. Phys. Chem. Chem. Phys. 2013, 15, 6945–6952. 10.1039/c3cp50738f. 23549450

[ref58] Yang J. J.; Strukov D. B.; Stewart D. R. Memristive devices for computing. Nat. Nanotechnol. 2013, 8, 13–24. 10.1038/nnano.2012.240. 23269430

[ref59] Lee J. S.; Lee S.; Noh T. W. Resistive switching phenomena: A review of statistical physics approaches. Appl. Phys. Rev. 2015, 2, 031303 10.1063/1.4929512.

[ref60] Cüppers F.; Menzel S.; Bengel C.; Hardtdegen A.; von Witzleben M.; Böttger U.; Waser R.; Hoffmann-Eifert S. Exploiting the switching dynamics of HfO_2_-based ReRAM devices for reliable analog memristive behavior. APL Materials 2019, 7, 091105 10.1063/1.5108654.

[ref61] Ilyas N.; Li D.; Li C.; Jiang X.; Jiang Y.; Li W. Analog Switching and Artificial Synaptic Behavior of Ag/SiO_*x*_:Ag/TiO_*x*_/p++-Si Memristor Device. Nanoscale Res. Lett. 2020, 15, 30 10.1186/s11671-020-3249-7. 32006131 PMC6994582

[ref62] Fernandez-Guillen I.; Aranda C. A.; Betancur P. F.; Vallés-Pelarda M.; Momblona C.; Ripolles T. S.; Abargues R.; Boix P. P. Perovskite Thin Single Crystal for a High Performance and Long Endurance Memristor. Adv. Electron. Mater. 2024, 10, 2300475 10.1002/aelm.202300475.

[ref63] Häusser M. The Hodgkin-Huxley theory of the action potential. Nature Neuroscience 2000, 3, 1165–1165. 10.1038/81426. 11127828

[ref64] Wilson H. Spikes, Decisions, and Actions: The Dynamical Foundations of Neuroscience; Oxford University Press, 1999.

[ref65] Hopper A. J.; Beswick-Jones H.; Brown A. M. A color-coded graphical guide to the Hodgkin and Huxley papers. Advances in Physiology Education 2022, 46, 580–592. 10.1152/advan.00178.2022. 36007940

[ref66] Robin P.; Kavokine N.; Bocquet L. Modeling of emergent memory and voltage spiking in ionic transport through angstrom-scale slits. Science 2021, 373, 687–691. 10.1126/science.abf7923. 34353953

[ref67] Katiyar R. K.; Sharma Y.; Barrionuevo Diestra D. G.; Misra P.; Kooriyattil S.; Pavunny S. P.; Morell G.; Weiner B. R.; Scott J. F.; Katiyar R. S. Unipolar resistive switching in planar Pt/BiFeO_3_/Pt structure. AIP Advances 2015, 5, 037109 10.1063/1.4914475.

[ref68] Kang K.; Ahn H.; Song Y.; Lee W.; Kim J.; Kim Y.; Yoo D.; Lee T. High-Performance Solution-Processed Organo-Metal Halide Perovskite Unipolar Resistive Memory Devices in a Cross-Bar Array Structure. Adv. Mater. 2019, 31, 1804841 10.1002/adma.201804841. 30932266

[ref69] Khurana G.; Kumar N.; Chhowalla M.; Scott J. F.; Katiyar R. S. Non-Polar and Complementary Resistive Switching Characteristics in Graphene Oxide devices with Gold Nanoparticles: Diverse Approach for Device Fabrication. Sci. Rep. 2019, 9, 1–10. 10.1038/s41598-019-51538-6. 31641183 PMC6806005

[ref70] Nardi F.; Balatti S.; Larentis S.; Ielmini D. Complementary switching in metal oxides: Toward diode-less crossbar RRAMs. Technical Digest - International Electron Devices Meeting, IEDM 2011, 709–712.

[ref71] Kumar S.; Williams R. S.; Wang Z. Third-order nanocircuit elements for neuromorphic engineering. Nature 2020, 585, 518–523. 10.1038/s41586-020-2735-5. 32968256

[ref72] Ramirez P.; Portillo S.; Cervera J.; Bisquert J.; Mafe S. Memristive arrangements of nanofluidic pores. Phys. Rev. E 2024, 109, 044803 10.1103/PhysRevE.109.044803. 38755814

