# Supplementary material for: Kinetics of Volatile and Nonvolatile Halide Perovskite Devices: The Conductance-Activated Quasi-Linear Memristor (CALM) Model
Source: J Phys Chem Lett. 2024 Dec 19;16(1):69–76. doi: 10.1021/acs.jpclett.4c03132 (PMC11726628; doi:10.1021/acs.jpclett.4c03132)
Supplement: Supplementary file 1 — jz4c03132_si_001.pdf [file jz4c03132_si_001.pdf]

Supporting Information

Kinetics of Volatile and Non-Volatile Halide Perovskite Devices: The Conductance-Activated Quasi-Linear Memristor (CALM) Model

Agustín Bou*,[1,2] Cedric Gonzales,[3] Pablo P. Boix,[4] Yana Vaynzof,[1,2] Antonio Guerrero,[3] Juan Bisquert*[4]

[1] Chair for Emerging Electronic Technologies, Technical University of Dresden, Nöthnitzer Str. 61, 01187 Dresden, Germany

[2] Leibniz-Institute for Solid State and Materials Research Dresden, Helmholtzstraße 20, 01069 Dresden, Germany

[3] Institute of Advanced Materials (INAM), Universitat Jaume I, 12006 Castelló, Spain.

[4] Instituto de Tecnología Química (Universitat Politècnica de València-Agencia Estatal Consejo Superior de Investigaciones Científicas), Av. dels Tarongers, 46022, València, Spain.

Corresponding authors: a.bou.catala@ifw-dresden.de, jbisquer@itq.upv.es

Experimental Details

Device Fabriction

The fluorine-doped tin oxide (FTO) substrates (TEC15) were partially etched with zinc powder and a 2 M hydrochloric acid solution. The etched samples were individually brushed to mechanically remove the residues of the etching process. The brushed samples were then subjected to a sequence of 15-minute sonication in deionized water with Hellmanex detergent solution, acetone, and isopropyl alcohol. The cleaned samples were blow-dried using a nitrogen gun.

Prior to the deposition of the poly(3,4-ethylenedioxythiopene) polystyrene sulfonate (PEDOT:PSS), the cleaned samples were subjected to an ultraviolet-ozone (UV-O_3) treatment for 15 minutes to further remove organic contamination on the surface and improve the surface wetting. The PEDOT:PSS solution (Clevios P VP. Al 4083) was filtered using a 0.45 µm Nylon filter and was statically spin coated onto the etched FTO substrates for 30 s at 3000 RPM with an acceleration of 1000 RPM/s. The deposited PEDOT:PSS was annealed at 100 °C for 5 minutes then the samples were immediately transported into a nitrogen-controlled glove box in preparation for the $MAPbBr_3$ perovskite deposition.

A 1.4 M $MAPbBr_3$ precursor solution was prepared using $PbBr_2$ (>98%, TCI), and MABr (>99.99%, Greatcell Solar) in 1:4 dimethylsulfoxide (DMSO) (≥99.9%, Sigma Aldrich):N,N-dimethylformamide (DMF) (99.8%, Sigma Aldrich) solution. A 50 µL $MAPbBr_3$ perovskite solution was statically spin coated onto the PEDOT:PSS layer via a

two-step antisolvent method: 10 s at 1000 RPM, followed by 40 s at 4000 RPM. A 100 µL toluene (99.8%, Sigma Aldrich) antisolvent was injected 32 s before the spin coating ended. The samples were then annealed at 100 °C for 30 minutes.

For samples with Ag/Au top contact configuration, a 15 nm Ag contact is initially thermally evaporated using a commercial Oerlikon Leybold Univex 250. Finally, an 85 nm gold contact was thermally evaporated as the top contact.

Voltage-dependent Transient Current Response

The characteristic $I-V$ curves of the perovskite devices were measured inside a nitrogen-controlled glove box under dark conditions using an Autolab PGSTAT204 with a scan rate of 1 V s^{-1}. The voltage-dependent transient current response of the device at an applied volage (V_{app}) pulses were measured using 20 voltage pulses with a pulse width (t_{app}) of 10 ms.

Internal Variable and Kinetic Time Constant Functions

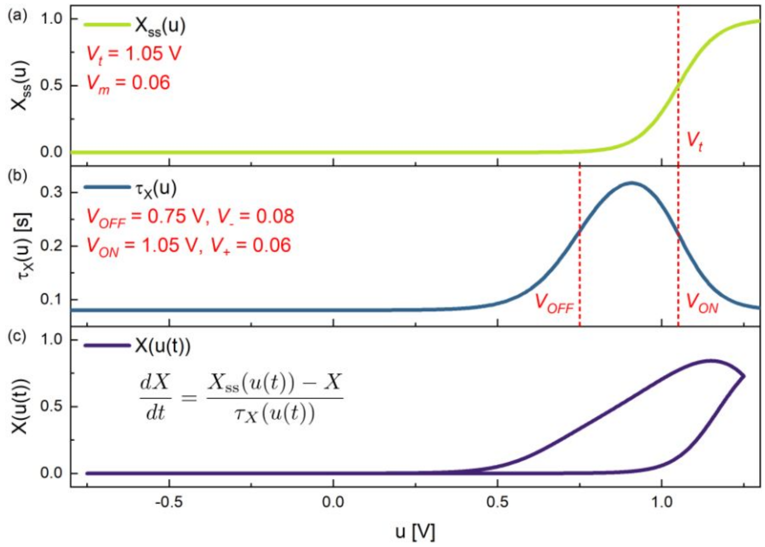

Figure S1. The corresponding (a) internal state variable function $X_{ss}(u)$, (b) the time constant function $\tau_X(u)$, and (c) the time-dependent internal state function $X(u(t))$ of the simulated characteristic $I-V$ response of Fig. 3a with the voltage and ideality factor parameters indicated.

Model Parameter Lists of Simulated Characteristic I-V Response

Table S1. The model parameter list of the simulated characteristic $I-V$ curves in Figs. 3 and 4 that emulate the volatile, nonvolatile gradual, and nonvolatile abrupt resistive switching of perovskite-based memristors.

**Parameters**	**Volatile**<br>*(Fig. 3 and Fig. 4a)*	**Nonvolatile Gradual**<br>*(Fig. 4b)*	**Nonvolatile Abrupt**<br>*10.1021/acs.jpclett.2c03669*<br>*(Fig. 4c)*

C_m (μF)	2	10	10
g_L (μS)	20	20	250
g_H (S)	0.01	0.01	0.01
V_t (V)	1.05	0.6	0.6
V_m (V)	0.06	0.05	0.01
$X_{SS,\,min}$	10^{-6}	10^{-6}	10^{-6}
τ_{max} (s)	0.3	10	10
V_{OFF} (V)	0.75	0.3	-0.5
V_- (V)	0.08	0.1	0.001
V_{ON} (V)	1.05	0.6	0.6
V_+ (V)	0.06	0.05	0.01
τ_{min} (s)	0.08	0.1	0.1

Table S2. Parameter list of the simulated characteristic $I-V$ curves in Figs. S2 and S3.

**Parameters**	**W/SiGe/a-Si/Ag** *10.1021/nl203687n* *(Fig. 5c)*	**MgOx:Ag** *10.1038/nmat4756* *(Fig. 5d)*	**SiOxNy:Ag** *10.1038/nmat4756* *(Fig. 5d)*	**HfOx:Ag** *10.1038/nmat4756* *(Fig. 5d)*	**p++ Si/SiO2/ASTO/Ag** *10.1021/acs.jpclett.1c03912* *(Fig. 6c)*	**SrRuO3/SrZrO3/Au** *10.1063/1.126902* *(Fig. 6d)*
C_m (F)	1×10^{12}	1×10^{12}	1×10^{12}	1×10^{12}	1×10^{9}	1×10^{9}
g_L (S)	2×10^{-12}	2×10^{-9}	2×10^{-9}	2×10^{-14}	0.00002	0.00002
g_H(S)	0.00001	0.00001	0.001	0.01	0.001	0.0002
V_t (V)	2	0.3	0.3	0.2	2.75	0.5
V_m (V)	0.005	0.001	0.001	0.001	0.3	0.001
$X_{SS,\,min}$	0.000001	0.000001	0.000001	1×10^{-9}	0.000001	0.000001
τ_{max} (s)	100	100	100	100	100	100
V_{OFF} (V)	0.5	0.01	0.01	0.01	-2.5	-0.5
V_- (V)	0.05	0.005	0.005	0.005	0.2	0.0001
V_{ON} (V)	2	0.3	0.3	0.2	2.75	0.5
V_+ (V)	0.005	0.001	0.001	0.001	0.3	0.001
τ_{min} (s)	0.1	0.01	0.01	0.001	0.1	0.1

Volatile and non-volatile memristors

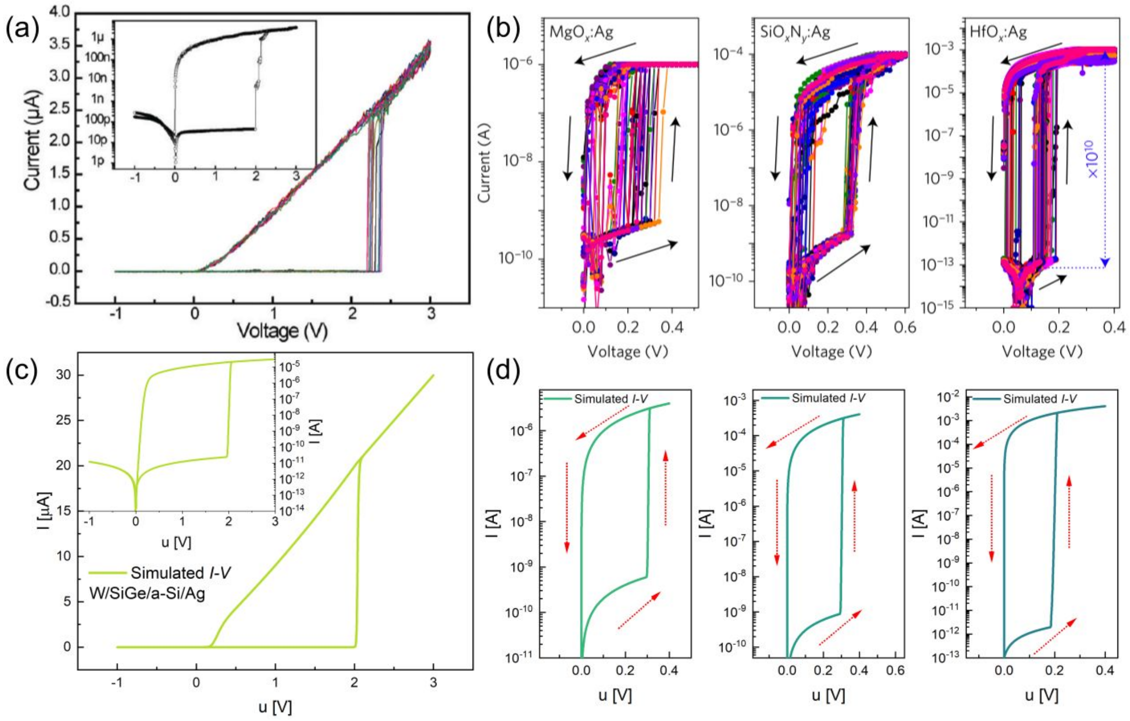

Figure S2. The (a) characteristic $I-V$ response exhibiting a threshold volatile switching of a W/SiGe/a-Si/Ag integrated on top of a CMOS chip with the (c) corresponding simulated curves using the model. Adapted with permission from Kim, K. H., Gaba, S., Wheeler, D., Cruz-Albrecht, J. M., Hussain, T., Srinivasa, N. & Lu, W. A functional hybrid memristor crossbar-array/CMOS system for data storage and neuromorphic applications. *Nano Letters* **12**, 389–395 (2012). Copyright 2012 American Chemical Society. The (b) characteristic $I-V$ curves of various oxide-based memristors with Ag exhibiting threshold volatile memories with the (d) corresponding simulated curves using the model. Reproduced with permission from Wang, Z., Joshi, S., Savel'ev, S. E., Jiang, H., Midya, R., Lin, P., Hu, M., Ge, N., Strachan, J. P., Li, Z., Wu, Q., Barnell, M., Li, G. L., Xin, H. L., Williams, R. S., Xia, Q. & Yang, J. J. Memristors with diffusive dynamics as synaptic emulators for neuromorphic computing. *Nature Materials* **16**, 101–108 (2017). Copyright 2017 Springer Nature.

Discussion Fig. S2

We show the threshold volatile switching of a high-density fully operational W / SiGe / amorphous-Si / Ag memristor crossbar-array / CMOS system by Kim et.al. that can reliably store complex binary and multilevel 1600 bitmap images (Fig. S2a).[1] By using the biological model, the characteristic $I-V$ curve is reproduced both in the linear and the semi-log scales (Fig. S2c) with the corresponding model parameters listed in Table S2. Most notably, the current beyond the SET voltage features a fully ohmic region which linearly increases with voltage. This ohmic response is due to the device maintaining its

high conductance state ($g_L + g_H$) at voltages above this threshold SET voltage as previously discussed.

Another material platform exhibiting volatile threshold switching are diffusive Ag-in-oxide-based memristors by Wang et.al. emulating both the short- and long-term plasticity of biological synapses (Fig. S2b).[2] The different oxide-based memristors emulate the biological Ca^{+} ion channel dynamics due to the diffusion of Ag metal atoms and spontaneous nanoparticle formation.[2] The biological model is able to simulate the stable resistive switching of the three different oxide materials with of 4 to 10 orders of magnitude ON/OFF ratios (Fig. S2d) with the corresponding model parameters tabulated in Table S2. Valuable insight on the intrinsic properties of the memristor stack can be extracted from the model parameters that would allow direct mechanistic analog with their synaptic functions.

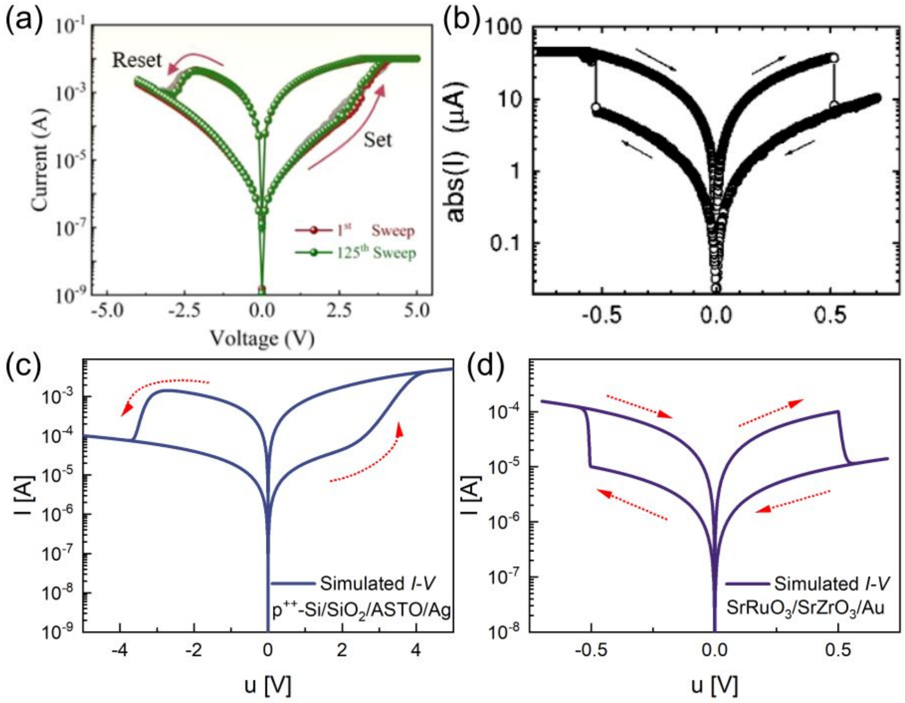

Figure S3. The (a) characteristic $I - V$ response of a p++-Si / SiO_2 / ASTO / Ag memristor device exhibiting a bipolar nonvolatile gradual switching with the (c) corresponding simulated $I - V$ response using the model. Adapted with permission from Ilyas, N., Li, C., Wang, J., Jiang, X., Fu, H., Liu, F., Gu, D., Jiang, Y. & Li, W. A Modified SiO_2-Based Memristor with Reliable Switching and Multifunctional Synaptic Behaviors. *Journal of Physical Chemistry Letters* **13**, 884–893 (2022). Copyright 2022 American Chemical Society. The (b) characteristic $I - V$ response of a $SrRuO_3$ / Cr-doped $SrZrO_3$ / Au memristor device exhibiting a bipolar nonvolatile abrupt switching with the (d) corresponding simulated $I - V$ response using the model. Reprinted from Beck, A., Bednorz, J. G., Gerber, C., Rossel, C. & Widmer, D. Reproducible switching effect in

thin oxide films for memory applications. *Applied Physics Letters* **77**, 139–141 (2000) with the permission of AIP Publishing.

Discussion Fig. S3

We next discuss memristors with bipolar nonvolatile resistive switching for binary in-memory applications, Fig. S3. The characteristic $I-V$ response of a p^{++}-Si / SiO_2 / Ag doped $SrTiO_3$ (ASTO) / Ag memristor device by Ilyas et.al. exhibits a nonvolatile gradual switching (Fig. S3a) and has been demonstrated to mimic simple learning and forgetting behavior in a 7×7 pixel array.[3] This gradual bipolar resistive switching can be simulated by having negative V_{OFF} and positive V_{ON} with relatively higher values of V_- and V_+ (Fig. S3c) as tabulated in the parameters list in Table S2. Similarly, a $SrRuO_3$ / Cr-doped $SrZrO_3$ / Au memristor by Beck et.al. has demonstrated reproducible abrupt bipolar switching with long term retention times for nonvolatile 2-bit storage (Fig. S3b).[4] Again, our biology-inspired model is able to reproduce this characteristic response similar to Fig. S3c but with lower values of V_- and V_+ (Table S2) resulting to the abrupt SET and RESET processes.

Experimental Voltage-Dependent Transient Current Response

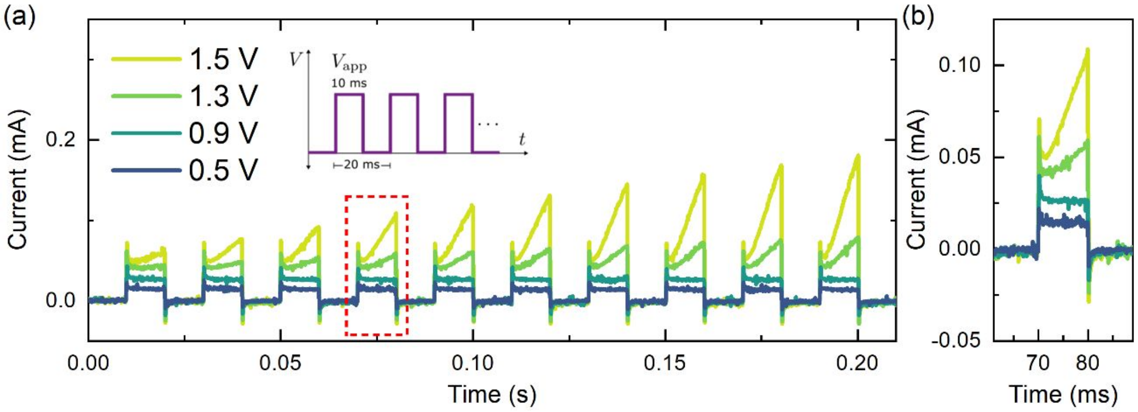

Figure S4. (a) The voltage-dependent transient current response of the FTO / PEDOT:PSS / $MAPbBr_3$ / Au perovskite memristor by applying 10 identical voltage pulses with an amplitude of V_{app} and a pulse width of 10 ms (schematic diagram shown in inset), (b) the magnified view of the transient current response of a single voltage pulse at representative V_{app} levels.

References

(1) Kim, K. H.; Gaba, S.; Wheeler, D.; Cruz-Albrecht, J. M.; Hussain, T.; Srinivasa, N.; Lu, W. A functional hybrid memristor crossbar-array/CMOS system for data storage and neuromorphic applications. *Nano Letters* **2012**, *12* (1), 389-395. DOI: 10.1021/nl203687n.

(2) Wang, Z.; Joshi, S.; Savel'ev, S. E.; Jiang, H.; Midya, R.; Lin, P.; Hu, M.; Ge, N.; Strachan, J. P.; Li, Z.; et al. Memristors with diffusive dynamics as synaptic emulators for neuromorphic computing. *Nat. Mater.* **2017**, *16* (1), 101-108. DOI: 10.1038/nmat4756.

(3) Ilyas, N.; Li, C.; Wang, J.; Jiang, X.; Fu, H.; Liu, F.; Gu, D.; Jiang, Y.; Li, W. A Modified SiO2-Based Memristor with Reliable Switching and Multifunctional Synaptic Behaviors. *Journal of Physical Chemistry Letters* **2022**, *13* (3), 884-893. DOI: 10.1021/acs.jpclett.1c03912.

(4) Beck, A.; Bednorz, J. G.; Gerber, C.; Rossel, C.; Widmer, D. Reproducible switching effect in thin oxide films for memory applications. *Applied Physics Letters* **2000**, *77* (1), 139-141. DOI: 10.1063/1.126902.
